# Sows’ Responses to Piglets in Distress: An Experimental Investigation in a Natural Setting

**DOI:** 10.3390/ani13142261

**Published:** 2023-07-10

**Authors:** Edoardo Collarini, Luca Capponcelli, Andrea Pierdomenico, Ivan Norscia, Giada Cordoni

**Affiliations:** Department of Life Sciences and Systems Biology, University of Torino (DBIOS), Via Accademia Albertina 13, 20123 Torino, Italy; edoardo.collarini@unito.it (E.C.);

**Keywords:** *Sus scrofa*, swine, domestic pig, vocalisation, isolation call, maternal behaviour, parental care

## Abstract

**Simple Summary:**

Maternal behaviour is a common trait in many mammalian species, and mothers can invest a lot of energy in parental care, ensuring a higher probability of survival for their infants. An interesting question is whether these behaviours are influenced by particular mechanisms and how the offspring can effectively attract the mother’s attention. In order to try to answer this question, we studied a group of sows reared in northern Italy (ethical farm Parva Domus, Cavagnolo, Turin) in which lactating and non-lactating females were present. Isolation calls emitted by the piglets were able to generate a state of anxiety in the mothers and can be therefore a valid example of a useful mechanism to request maternal care effectively. Furthermore, we not only observed that lactating females responded and reacted more to the vocalisations of piglets from other broods, but also that less aggressive mothers responded more. Finally, we found that certain vocalisation characteristics may influence the type of response by the mothers. Therefore, several factors seem to play a key role in eliciting response behaviour in sows.

**Abstract:**

Domestic pigs (*Sus scrofa*) possess complex socio-cognitive skills, and sows show high inter-individual variability in maternal behaviour. To evaluate how females—reared under natural conditions—react to the isolation calls of their own piglets or those of other females, we conducted observations and experimental trials. In January–February 2021, we conducted all-occurrences sampling on affiliation, aggression, and lactation (daily, 7:30–16:30 h) on six lactating and four non-lactating females at the ethical farm Parva Domus (Turin, Italy). The trials (30 s each, *n* = 37/sow) consisted of briefly catching and restraining a piglet. We recorded the sow response (none/reactive/proactive movement towards the piglet; self-directed anxiety behaviours such as body shaking) before and during the trial and under control conditions. Increased levels of anxiety behaviour in sows were accompanied by an increased frequency of responses. Less aggressive sows and lactating sows showed the highest frequencies of response. Finally, the isolation calls’ maximum intensity had an influence on the type of response observed, with higher proactive response frequencies following lower intensity isolation calls. Our results suggest that being under lactation could play a key role in increasing sow response levels and that specific acoustic features may influence the response.

## 1. Introduction

In mammals, the attachment between mother and infant develops during pregnancy or immediately after delivery, and it is promoted by social cues such as communicative signals, including vocalisations [1]. In different mammalian species, infants emit vocalisations when they are separated from their mothers or, more generally, when they are distressed to maintain or regain contact with their mother [2,3,4,5,6,7]. Distress acoustic signals—remarkably similar across mammals (e.g., isolation calls)—may convey information on the emitter’s conditions (e.g., via intensity or frequency) [8,9]. Distressed and endangered infants may elicit an increase in mothers’ arousal, induce mothers to increase vigilance, and establish communication with their offspring for protection [2,5,7,8,9,10,11,12,13,14,15,16,17,18,19,20,21,22,23,24,25,26,27]. In sows, hormone stress levels can influence maternal behaviour [28], although it is unclear how transient arousal (e.g., measured via anxiety behaviours) can affect maternal behaviour under natural conditions.

Pigs show behaviours that are specifically associated with physiological anxiety (scratching, head-body rubbing, head shaking, yawning, and vacuum chewing) [29]. Pigs have a large vocal repertoire [30,31,32], and acoustic communication is often used in mother–infant interactions [33,34,35], especially during early interactions, such as lactation and abrupt separations [36,37]. Piglets vocalise more when they need maternal care and show a context-dependent plasticity of acoustic signals [38,39], screaming when they perceive an imminent threat [40], and emitting different vocalisations depending on the situation. For example, distress vocalisations are emitted by piglets when they are isolated from their mother, when they need heat, or if they feel pain [37,38,39,40,41,42]. In particular, isolation calls emitted by piglets are characterised initially by quiet, low-frequency sounds and later by louder, high-frequency sounds [37,38,39,40,41,42,43]. In playback experiments, it has been documented that sows (i) are able to recognise their offspring depending on the acoustic characteristics of the piglet calls that are typical of the litter [37]; and (ii) can show stronger responses to the acoustic signals of infants in distress, especially when the duration and intensity of piglet isolation calls are higher [41].

Different studies carried out under experimental conditions have shown that the maternal response of sows may depend not only on the acoustic properties of isolation calls (perceptual factors) but also on individual and social factors. Specifically, female pigs may develop stable social relationships, especially in the wild, and lactating sows can show allomaternal care in response to the piglets of other, especially strongly related, sows [42,43,44]. On the other hand, under experimental conditions, it has been observed that sows can respond more intensely to the playback of isolation calls when the acoustic signal is emitted by their own litter [41]. Moreover, under experimental conditions, less aggressive sows can show stronger maternal behavioural responses [45].

Despite the above information, no study so far has specifically addressed the relation between arousal increase and responses of females (both mothers and non-mothers) under natural conditions in pigs and how such responses can be modulated by individual, perceptual, and social factors (considered together).

To fill this gap, based on the above pieces of evidence on pigs, we formulated the general hypothesis that sows may respond to piglets’ isolation calls via arousal increase, and that their response may vary depending on individual (i.e., aggressive levels, being a lactating sow/mother sow or not), perceptual (i.e., call intensity and duration), and social factors (i.e., relationship between mothers and other sows).

## 2. Materials and Methods

### 2.1. Study Site and Subjects

**Figure 1:**
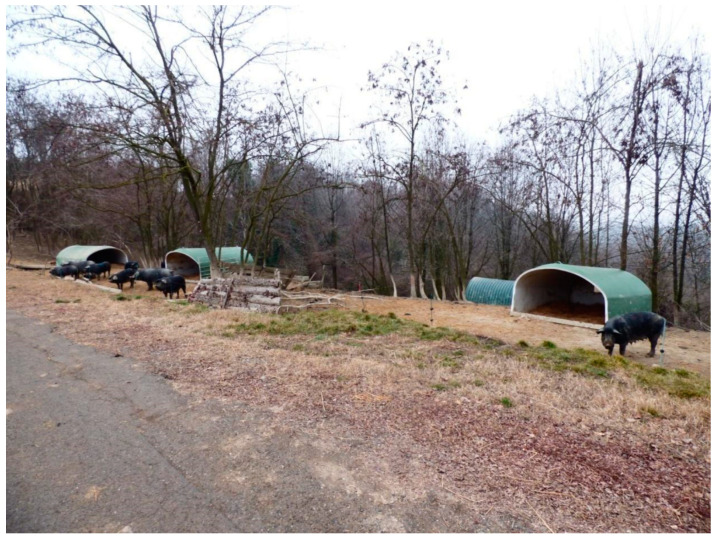
Sow shelters in the study area.

This study was carried out in January–February 2022 (temperature: average = 3.9 °C ± 0.4; min = −0.6 °C ± 0.4; max: 11.6 °C ± 0.7; humidity: 74.3% ± 2.2) at the “Ethical Farm Parva Domus”, in Cavagnolo, Turin (Italy). The sows under study (Parma Black breed) could freely move and forage in a 1.0 ha area of natural grassland/woodland habitat. Four shelters that could accommodate multiple females were available in the upper part of the outdoor area, spaced a few metres apart (Figure 1).

The animals were provisioned with food (Ciclo Unico P, SILDAMIN©, Pavia, Italy) every day from 8:30 to 10:30 in a feeding zone (upper part of the area); water was available ad libitum. Sows could integrate their diet with roots and leaves that they could find in the environment. For individual recognition, all the individuals of the group, including piglets, were marked with the non-toxic spray Raidex© for livestock or identified based on unique distinctive features (e.g., a combination of colour, black or white spots, wattles, etc.). To estimate weight, we carried out a set of size measurements, including body length and chest girth, at the beginning of the data collection period. Body length and chest girth can explain around 90% of the weight variation and can therefore provide a reliable estimate of pig live weight [46]. The measurements were taken with the aid of a clothing tape while the subjects were feeding in a spot (with no necessity to constrain them). Body length was measured from the neck base to the tail base right above the shoulder, and chest girth was determined by measuring the circumference of the chest area behind the forelegs [47]. Weight (kg) was estimated by applying the following formula [46]:

0.39 × length + 0.64 × girth.

**Figure 2:**
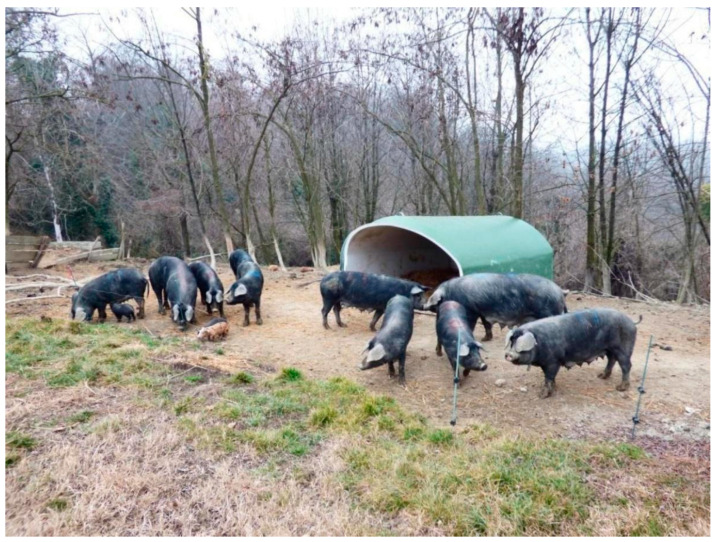
The study group (one individual is not visible).

All sows (Parma Black breed; estimated average weight, lactating females: 122.03 Kg ± 4.83; non-lactating females: 110.89 ± 3.43) were sexually mature (with sexual maturity being reached at 5–6 months of age; [48]). The group was composed of 6 lactating females (1 to 1.5 years old; 3 primiparous and 3 non-primiparous, who had experienced at least one previous delivery) and 4 non-lactating females (7 months old). All sows were already habituated to the human presence, so it was possible to easily approach and mark them from the beginning of the study. However, the first five days were used for individual identification and training on observational data collection (Figure 2).

All the lactating sows had given birth within a period of 24 days. One sow (SR) lost her litter on the first day after birth (because the piglets had been inadvertently crushed by another female, as observed during the second day on the field) but kept lactating piglets from other litters throughout the study period. No sows showed stereotypic behaviour (e.g., repeated and/or abnormal behaviours in the absence of any perturbing event). All the sows had the same father and could be either full-siblings or half-siblings. Table 1 shows the details of the sows under study, including the date of delivery.

### 2.2. Data Collection and Operational Definitions

Behavioural data collection was carried out on a daily basis from around 7:30 a.m. to 4:30 p.m. Around 80% of the time, sows would stay in the area surrounding the shelters, so it was possible to observe them easily all together or in subgroups. Affiliation states (sensu Altmann [49]; lay-in-contact and sit-in-contact) and suckling session data were gathered via audio and/or video recording by using the *all-occurrences sampling* method [49]. The observers noted which sows were not visible during observations to be able to calculate the observation time of each sow and dyad. Individual behavioural frequencies were determined as the number of observed behavioural bouts for a given individual normalised over the observation time of that individual. The dyadic interaction frequencies for each pair of sows were determined as the number of interactions exchanged normalised over the observation time of the dyad (when the sows of the dyad could be both observed at the same time). The dyadic affiliation frequencies were used as indicators of the quality of the relationship between sows.

Each day, we recorded all the conflicts occurring right after food provisioning by the farmer over a 15 min time window. For each aggression, the identities of the aggressor (sow directing the first aggressive pattern to another sow) and the recipient (sow receiving the first aggressive pattern) were noted. The behaviours were categorised following the ethogram used by Norscia et al. (2021) [29] and Cordoni et al. (2022) [50] and described in Table 2. Ethological observations were stopped when other activities were carried out (e.g., experimental trials, feeding). Observers stayed around 10 m from the piglets and sows.

### 2.3. Experimental Trials

**Figure 3:**
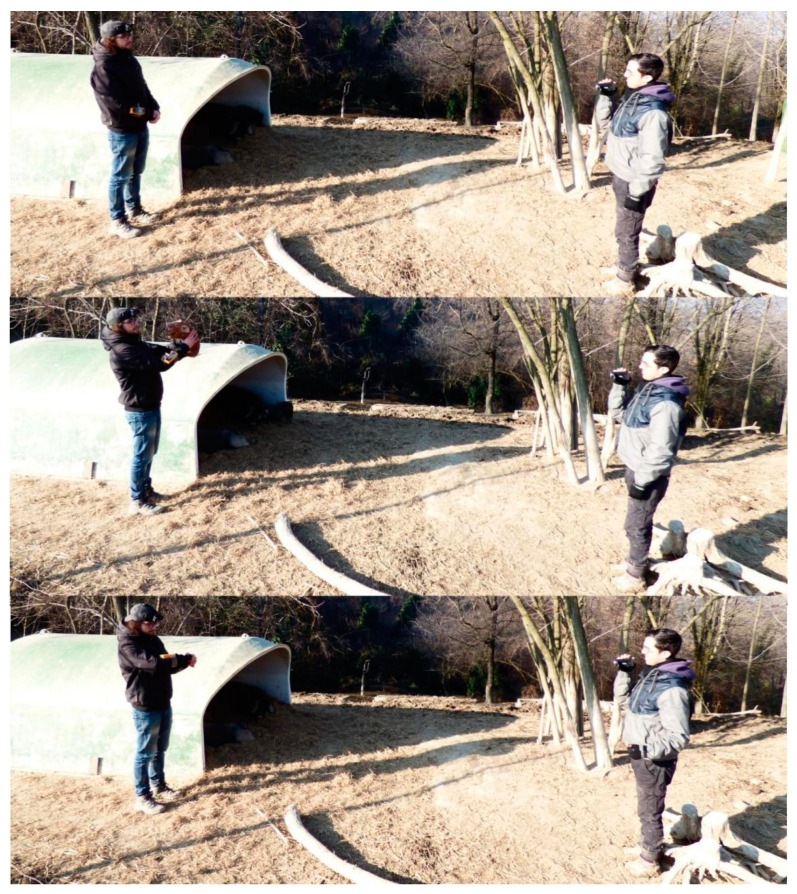
Trial sequence (**top**: pre-trial; **middle**: trial phase; **bottom**: MC).

The experimental sessions were carried out between around 12:30 and 16:30 p.m. in similar environmental conditions (no rain and in the shelter area). The experimenters wore the same external clothes across trials (Figure 3). To ensure that the sows were not in an arousal state and allow cross-individual and cross-trial comparison, the starting condition of each trial session was the following: at least one mother with litter and one non-mother had to be present within the shelter area (≤15 m), with sows inactive (but awake), in the absence of external perturbing events. At the time of the trials, all individuals were in the shelters or around (≤30 m) and not dispersed. The experimenter was always the same (L.C.) and would take position within 1 m from the shelter where at least one piglet was found. The first videographer—always the same (A.P.)—would take a position three metres away from the experimenter, directly facing him. A second videographer (I.N.) would take a position within 10 m of the other videographer when necessary and would record the sows that the first videographer could not film (off-screen sows). Distances, including the distance between the vocalising piglet and sow, were predetermined via measuring tape and barricade stripe tape (used to mark metres) to calculate the metres between shelters and between experimenters and animals. To avoid possible habituation issues, each sow was tested a maximum of three times per day.

The experimental session was composed of the following phases (Figure 3): (i) pre-trial phase (PRE): the experimenter and the videographers stayed still for 30 s; (ii) trial phase (TP): the experimenter caught and restrained a piglet (for max 30 s) and the sow behaviour was recorded during the period following the beginning of piglet isolation calls (characterised initially by quiet, low-frequency sounds and later by louder, high-frequency sounds); (iii) matched-control (MC): for each TP, a corresponding 30 s matched-control (MC) observation of the behaviour of the same individuals was recorded on the next possible day, in the same social and environmental context, in the absence of external perturbing events. The PRE and MC phases were used as controls to ensure that the sow behavioural change (if any) was a reaction to the isolation call emitted by the piglet and not enacted regardless. In PRE, TP, and MC, we collected data on self-directed behaviour with a previously demonstrated association with anxiety (self-scratching, body rubbing, head/body shaking, yawning, vacuum chewing; Table 2; [29,50]). During TP, the maximum intensity of the isolation call (dB) was recorded using a phonometer (V-RESOURCING RZ1358; measuring range: 30–130 dBA, accuracy within +/−1.5 dB; resolution: 0.1 dBA) positioned at a distance of 50 cm from the piglet emitting the vocalisation (emitter). To ensure data independence, the inter-trial interval was at least 3 min (the time that is required for the females to return to homeostasis as per Norscia et al., 2021), and a new trial was carried out when all the sows were again in the starting condition (see above).

The trial was considered as null if at least one of the following conditions occurred: (i) an isolation call was emitted from a subject other than the piglet caught and restrained by the experimenter; (ii) no isolation call occurred; (iii) a disturbance factor emerged (e.g., car or tractor passing close by, dog barking, etc.); (iv) if the sow moved their head or body when the experimenter was in the process of catching the piglet, before the isolation call occurred (trial invalid for the disturbed sow only). In no case the piglets showed any of the anxiety behaviours reported in Table 2 after the ‘catching’ phase.

In the field, we randomly selected the sow for each trial by blindly picking the name from a pool of all sows’ names (a sow name was removed from the pool when three trials were reached for that day). Trial validity was checked along the way to keep track of the valid trials. We carried out 37 valid sessions/sow out of a total of 108 sessions. In detail, for the different categories, we carried out 12 valid sessions/lactating sow, with the sow being the mother of the vocalising piglet, and 25 valid sessions/lactating sow, with the sow as a non-mother.

### 2.4. Operational Definitions, Video Coding

We recognised three categories of sows: non-lactating females (gilts), lactating females that were not the mothers of the vocalising piglets (hereafter, lactating non-mothers), and mothers of the vocalising piglets (hereafter, lactating mothers). We identified three possible types of sow behavioural responses, extracted from videos: no response (the sow did not move at all), reactive responses (the sow moved her head towards the experimenter or changed position, directing her body towards the experimenter, but did not leave the spot where she stayed before the screaming), and proactive response (the sow moved towards the experimenter). In no case did the sows move away from the vocalising piglet.

Because it was not possible to predict when and for how long the piglet would vocalise in TP, for cross-phase behavioural comparisons, we normalised the response bouts over the observation seconds/phase across trials. The isolation call duration (Mean ± SE: 10.13 s ± 8.18) was extracted from the trial videos to be tested as a possible variable affecting the sow reaction (see the ‘Statistical analyses’ section below).

The isolation call intensity was then recalculated from the maximum vocalisation intensity recorded close to the vocalisation emitter, compared to the attenuation provided by the law of the inverse distance [51,52] on the basis of the distance between the experimenter and the sow (vocalisation receiver), by using the formula (L_eq_ = vocalisation decibels for the receiver; L_rif_ = vocalisation decibels recorded at 50 cm to the emitter; r = linear distance between emitter and receiver; r_rif_ = distance to the emitter):

L_eq_ = L_rif_ − 20 × Log_10_ (r/r_riff_)

The sex and age of the piglet and the distance between the vocalising piglet and the tested sow were considered control variables.

The extraction of behaviours (Table 2) from videos was carried out via the freeware Potplayer 1.7.21. In particular, slow motion and/or frame-by-frame analysis were applied to extract data on sow responses and anxiety behavioural events (sensu Altman, 1974 [49]). The video coding on affiliation states, conflict and anxiety events was carried out by A.P. and L.C., and the inter-observer reliability was determined on 10% of videos based on Cohen’s k for aggression, anxiety behaviours, and affiliative states (Cohen’s 0.87 ≤ k ≤ 0.92). The coding of sow responses (reactive, proactive, or no response) was considered valid only in the case of 100% agreement across three observers (A.P., L.C., and E.C.) for all trials.

### 2.5. Statistical Analyses

To verify the variation of sow behaviour (anxiety behaviours and behavioural changes at the individual level) across phases (PRE, PT, and MC), we applied a non-parametric Friedman’s test for k dependent samples (non-normal data distribution of data; Kolmogorov–Smirnov test: N_sows_ = 10; 0.001 ≤ *p* ≤ 0.094). We applied the Dunn post hoc test for pairwise comparisons, with the significance level of probability adjusted downward using the Bonferroni correction.

We ran a first GLMM (GLMM_1_) to verify what individual factors affected the probability of observing sow response (target variable: presence/absence of response) to the piglet isolation call (N_cases_ = 370). We tested the following fixed factors: (i) sow category (non-lactating female, lactating non-mother, lactating mother); (ii) sow aggressive level (non-top/top quartile). As further control fixed factors we included: (i) piglet age (days); (ii) piglet sex (male/female); (iii) distance between the vocalising piglet and sow (metres); (iv) isolation call duration category (non-top/top quartiles); (v) max decibel intensity of isolation call (non-top/top quartile); (vi) sow affiliation level (non-top/top quartile); (vii) number of trials for each sow (numeric). The sow and piglet identities were included as random factors.

The second GLMM (GLMM_2_) was restricted to the cases where a response from the sow was observed (N_response_cases_ = 58), to test what perceptive factors can influence the type of response. The target, dependent variable was the type of sow response (proactive if the sow moved towards the experimenter; reactive if the sow moved her head towards the experimenter or changed position directing her body towards the experimenter but did not leave the spot) to the piglet’s isolation call. We tested the main effect of the following fixed factors: (i) isolation duration category (factor: 0 = non-top quartiles; 1 = top quartile); and (ii) max decibel intensity of vocalisation (factor: 0 = non-top quartiles; 1 = top quartile). The sow and piglet identities were included as random factors.

The third GLMM (GLMM_3_) was carried out on the sows that were not the mother of the vocalising piglet (N_non_mother_cases_ = 320) to test whether the relationship between the sow and the mother of the vocalising piglet would influence the response probability. The target, dependent variable was the occurrence of the response (presence/absence) of the sow to the piglet’s isolation call. We tested the main effect of the following fixed factors: (i) aggressive levels of the dyad non-mother and mother of the vocalising piglet (factor: 0 = non-top quartiles; 1 = top quartile); and (ii) affiliation levels of the same dyad (factor: 0 = non-top quartiles; 1 = top quartile). The sow and piglet identities were included as random factors.

The last GLMM (GLMM_4_) was carried out on lactating females only (N_lactating_female_cases_ = 222) to check whether lactation variables would influence the response. The target, dependent variable was the occurrence of the response (presence/absence) of the sow to the piglet’s isolation call. We tested the main effects of the following fixed factors: lactation duration (factor: 0 = non-top quartiles; 1 = top quartile); and lactation frequency (factor: 0 = non-top quartiles; 1 = top quartile). The sow and piglet identities were included as random factors.

The association between each GLMM analysis and specific assumptions derived from the general hypothesis are shown in Table 3.

We fit the GLMMs in R [53] by using the function “glmer” of the R-package lme4 [54], and we plotted the scaled (quantile) residuals for fitted (generalised) linear mixed models (Figure S1 in Supplementary Materials) by using the R-package DHARMa [55]. We established the significance of the full model by comparison to a null model that only included the random effects [56]. We used a likelihood ratio test [57] to test this significance (ANOVA with argument ‘Chisq’). We calculated the *p* values for the individual predictors based on likelihood ratio tests between the full and the null models by using the R-function “drop1” [58]. As the target variables were binomial, a binomial error distribution was used. For significant multinomial predictors, we performed all pairwise comparisons with the Tukey test [59] using a multiple contrast package (multcomp). We reported the Bonferroni-adjusted *p* values, estimate (Est), standard error (SE), and Z values.

We obtained the variance inflation factor (VIF) for the three numeric variables of GLMM_1_ (distance, piglet age, and trial number) via the “vif” function in R. All VIF values were >1.00, thus indicating no collinearity. We calculated the effect size via the package “effectsize” and the function effectsize, which returns the best effect-size measure for the provided input GLMM. For all tests (except in the case of Bonferroni correction, see above), the significance probability threshold was fixed at 0.05.

## 3. Results

**Figure 4:**
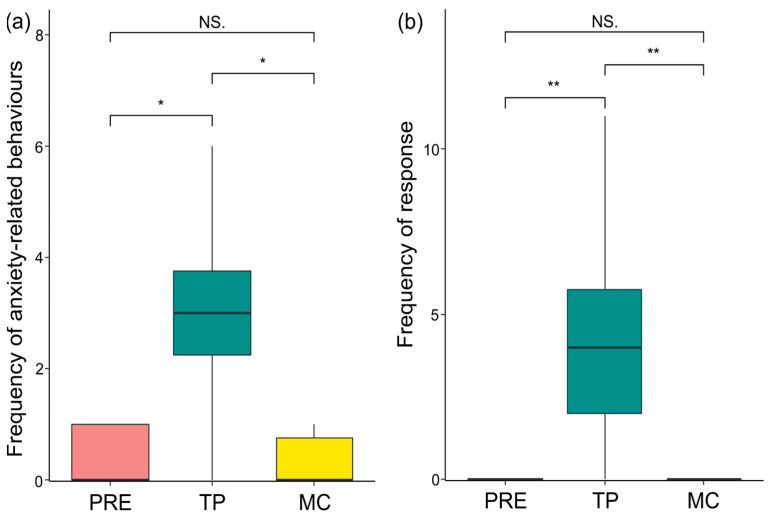
Box plot showing that (**a**) differences in levels of anxiety-related behaviours across PRE, TP, and MC (Friedman test: N_sows_ = 10, χ^2^ = 11.655, df = 2, *p* = 0.003; PRE vs. TP: *p* = 0.042; MC vs. TP: *p* = 0.030; PRE vs. MC: *p* = 1.000); (**b**) differences in levels of response behaviours across PRE, TP, and MC (Friedman test: N_sows_ = 10, χ^2^ = 18.000, df = 2, *p* < 0.001; PRE vs. TP: *p* = 0.008; MC vs. TP: *p* = 0.008; PRE vs. MC: *p* = 1.000). Horizontal line: median value; box: interquartile range; vertical line: minimum and maximum values in the data. NS = non-significant, * = *p* < 0.05; ** = *p* < 0.01.

There was a significant variation in the frequencies of anxiety behaviours of sows across the three phases (PRE, TP, MC; Friedman test: N_sows_ = 10, χ^2^ = 11.655, df = 2, *p* = 0.003). The pairwise comparisons (via Bonferroni–Dunn post hoc test) revealed a significant difference between PRE and TP (*p* = 0.042) and MC and TP (*p* = 0.030), but not between PRE and MC (*p* = 1.000). In particular, the sow showed the highest levels of anxiety behaviours in TP than in the PRE and MC phases (Figure 4a), thus confirming an anxiety increase following the piglet isolation call.

There was a significant variation in the behavioural response of sows across the three phases (PRE, TP, MC; Friedman test: N_sows_ = 10, χ^2^ = 18.000, df = 2, *p* < 0.001). The pairwise comparisons (via Bonferroni-Dunn post hoc test) revealed a significant difference between PRE and TP (*p* = 0.008) and MC and TP (*p* = 0.008), but not between PRE and MC (*p* = 1.000). In particular, the sow response was higher in the TP phase than in the PRE and MC phases (Figure 4b), thus indicating that such a response was likely associated with the vocalising piglets and not with other factors (e.g., the mere presence of experimenters).

**Figure 5:**
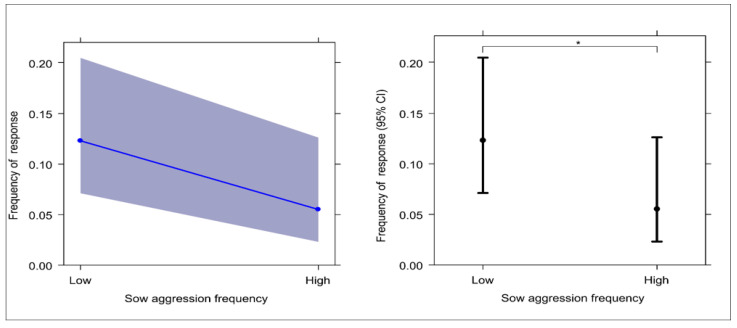
The effect plot (**right**) and the error bars (**left**) show the significant effect of sow aggression frequency on response frequency. (Likelihood ratio test: χ^2^ = 31.226; df = 10; *p* < 0.001). Effect plot: The slope of the blue line indicates the magnitude of the main effect. Error bars: 95% confidence interval (bars) around the mean (dots). * *p* < 0.05.

GLMM_1_ was carried out to verify the effect of individual factors on the target variable (the presence/absence of sow response). The full model (including all fixed factors and control fixed factors) and the null model (only including the random factor) significantly differ (likelihood ratio test: χ^2^ = 31.226; df = 10; *p* < 0.001). Because at least one predictor had a significant effect on the target variable, we applied the drop1 procedure. We found that the sow category and the aggression level of the sow had a significant main effect on the probability of observing a response, whereas the affiliation level of the sow and the other control fixed factors (distance, piglet age and sex, screaming duration, isolation call intensity, and trial number) did not (Table 4). In particular, sows that showed lower levels of aggression most likely responded to vocalising piglets (Table 4; Figure 5).

**Figure 6:**
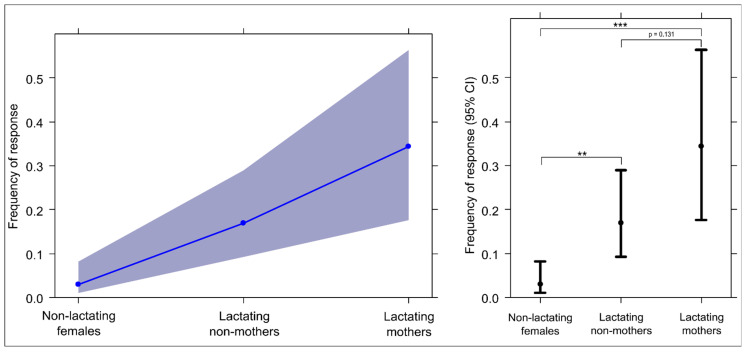
The effect plot (**right**) and error bars (**left**) show the effects of the “sow category” variable on response frequency (likelihood ratio test: χ2 = 31.226; df = 10; *p* < 0.001; Tukey test; lactating non-mothers vs. non-lactating females, *p* = 0.006; lactating mothers vs. non-lactating females, *p* < 0.001; lactating mothers vs. lactating non-mothers, *p* = 0.131). Effect plot: The slope of the blue line indicates the magnitude of the main effect. Error bars: 95% confidence interval (bars) around the mean (dots). ** = *p* < 0.01; *** = *p* < 0.001.

Moreover, lactating females (either mothers or non-mothers of the vocalising piglet) responded significantly more than non-lactating females (Table 4; Figure 6; Tukey test; lactating non-mothers vs. non-lactating females, Est = 1.912, SE = 0.630, Z = 3.037, *p* = 0.006; lactating mothers vs. non-lactating females, Est = 2.859, SE = 0.743, Z = 3.849, *p* < 0.001). Lactating mothers did not respond significantly more frequently than lactating non-mothers (Tukey test; Est = 0.947, SE = 0.497, Z = 1.905, *p* = 0.131). Hence, lactation (present or not) makes a major difference in the probability of observing a response by a sow to a vocalising piglet.

**Figure 7:**
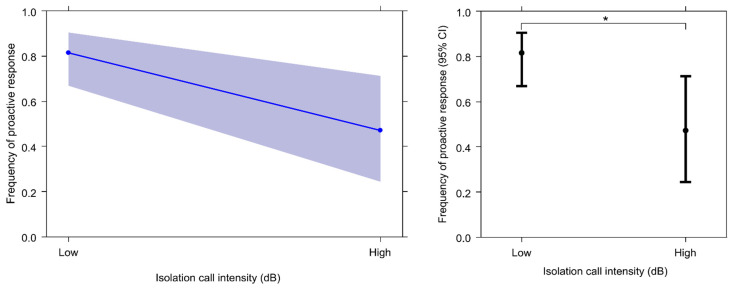
The effect plot (**right**) and the error bars (**left**) show the significant effect of the “isolation call intensity” variable on response frequency (likelihood ratio test: χ^2^ = 6.390, df = 2, *p* = 0.041). Effect plot: The slope of the blue line indicates the magnitude of the main effect. Error bars: 95% confidence interval (bars) around the mean (dots). * *p* < 0.05.

GLMM_2_ was carried out on response cases to verify whether isolation call duration or maximum intensity would significantly affect the type of sow response (reactive/proactive). The full model (including all fixed factors) and the null model (only including the random factor) significantly differ (likelihood ratio test: χ^2^ = 6.390; df = 2; *p* = 0.041). Because at least one predictor had a significant effect on the target variable, we applied the drop1 procedure. The maximum isolation call intensity, but not the duration, had a significant main effect on the type of response (Table 4), with a proactive response being most likely observed in cases of lower maximum decibels of the vocalisation (Figure 7). Hence, sows passively reacted to high-intensity vocalisations and most frequently moved towards the vocalising piglets when the maximum intensity of the isolation call was reduced.

GLMM_3_ was carried out on the lactating non-mothers and non-lactating females to verify whether the positive or negative relationship with the mother of the vocalising piglet (affiliation and aggression dyadic levels) would affect the probability of observing a response (presence/absence). The full model (including all fixed factors) and the null model (only including the random factor) did not significantly differ (likelihood ratio test: χ^2^ = 1.323; df = 2; *p* = 0.516). Hence, no predictor had a significant effect on the target variable.

GLMM_4_ was carried out on lactating females (mothers and non-mothers of the vocalising piglet) to verify whether lactation frequency and duration would affect the response probability (presence/absence). The full model (including all fixed factors) and the null model (only including the random factor) did not significantly differ (likelihood ratio test: χ^2^ = 0.325, df = 2, *p* = 0.850). Thus, in this case also, no predictor had a significant effect on the target variable.

## 4. Discussion

In this study, we found that (i) as it occurs in other social mammals [5,7,10,11,15,16,17,18,19,20,21,22,23,60,61,62,63], anxiety levels and response behaviour increased in tandem, both showing a peak when piglets emit the isolation call; (ii) lactating females (but not necessarily mothers) responded more to piglets in distress, in partial agreement with previous studies in mammals according to which mothers are the most responsive [64]; (iii) higher proactive response levels when isolation calls were characterised by low (rather than high) intensity, possibly because other acoustic features may interfere (such as amplitude and fundamental frequency [65]). Indeed, taken all together, these results are in line with the adaptive role that isolation calls or cries play in mammals. For example, in *Homo sapiens,* the crying of infants has signalling functions essential for their survival [2,10,18]. Among non-human primates, infants of common marmosets (*Callithrix jacchus*) emit their species-specific isolation calls (called “isolation phee”), and similar acoustic signals have been described in rhesus macaques (*Macaca mulatta*) to elicit mothers’ attention [66,67,68] and in strepsirhines [69]. Beyond primates, isolation calls are used to possibly get attention in bats of different species [70,71,72,73,74], guinea pigs [75], mice [76,77], kittens [78,79,80,81], reindeer [82], pinnipeds [83,84], and cetaceans [85,86]. Our results are discussed in detail below.

### 4.1. Sow Response and Anxiety Behaviours

As expected, we found increased levels of anxiety-related behaviour and increased frequency of response behaviour in the sows following the isolation calls of piglets (Figure 4a,b; assumptions 1a and 1b from Table 3 confirmed). Thus, the increase in the response frequency of sows may be related to an increase in anxiety levels, which could lead to increased vigilance. In line with previous literature [26,27,62,63], our results may suggest that a key role is played by increased vigilance in sows following hearing the isolation call emitted by piglets.

In social species, recognising stress signals emitted by other individuals can be highly adaptive [87]. Our findings could suggest that sows are able to recognise the distress of piglets and enact behavioural patterns that lead to distress reduction. This finding is in agreement with observations on other mammalian species—including rodents, domestic ungulates, and non-human and human primates-which indicate that acoustic signals emitted by immature subjects can provide distinctive and reliable indications of the offspring’s emotional state to attract the mother’s attention and elicit a rapid behavioural response [41,88,89,90,91,92,93,94,95,96,97,98,99]. In particular, our results are in line with previous studies on domestic pigs that have shown that piglets’ vocalisations may communicate their emotional state and attract the mother’s attention [41,89] and that individuals are sensitive to the internal states of others and can react to their distress [1,100,101,102]. Thus, considering our results, we can highlight that sows are able to react to the vocalisations of piglets experiencing distress when isolated and that in this species, the response of the sows to the acoustic signals may be decisive for the survival of the offspring.

Besides increased vigilance, which remains the most parsimonious explanation, sow responses might be related to affective arousal triggered by the detection of conspecifics’ distress. This phenomenon—known as emotional contagion—has been observed in several mammalian species, including the domestic pig [100]. In line with this hypothesis, this phenomenon can be particularly pronounced during maternal care, because mothers are emotionally affected by the state of their offspring [87]. In particular, in humans, mothers may experience physiological arousal when they witness their baby crying [20,25], and infant cries can be particularly stressful for mothers [103,104,105], requiring a rapid response to provide the necessary care to infants [20,106]. Further studies are necessary to investigate this aspect by comparing the reactions of sows to emotional and non-emotional stimuli.

Therefore, the results of this study seem in line with the evolutionary importance of the ability to recognise and react to distress signals in infants in order to allow a higher probability of offspring survival.

### 4.2. Individual, Perceptive, and Social Factors Affecting Sow Response

We expected that lactation and motherhood would enhance the sow response to piglet isolation calls. However, this expectation was not fully supported because the duration and frequency of lactation did not affect the response levels in sows (assumption 4 not confirmed), even though lactating females (not necessarily the mothers of the distressed piglets) responded more than non-lactating females (Figure 6, assumption 1c from Table 3 partially confirmed). This result may be explained by the fact that sows can show high inter-individual variability in the duration and frequency of lactation [107]. However, the response of the sows under study appeared to be mainly mediated by lactation per se rather than by its duration and frequency, possibly due to circulating pro-maternal hormones. Indeed, hormones implicated in delivery, lactation, and weaning (i.e., mostly prolactin and oxytocin) lead to an increase in care behaviours (e.g., retrieval, nursing, and grooming) and the formation of mother-infant attachment [108,109,110,111]. Interestingly, oxytocin may increase the salience of infant vocalisations by stimulating retrieval behaviour in mothers [112].

We expected a higher frequency of response to piglet isolation calls by sows showing lower levels of aggression. In agreement with this assumption, we found that the level of aggression had a significant effect, with less aggressive sows showing the highest levels of responses (Figure 5; assumption 1d from Table 3 confirmed). As concerns affiliate, we expected a higher frequency of response to piglet isolation calls by sows showing generally higher levels of affiliation and, more specifically, by sows sharing more affiliation with the mother of the vocalising piglet. Contrary to these assumptions, sow affiliation levels did not affect the response (assumptions 1e and 3 from Table 3 not confirmed). Hence, affiliation did not affect the sow response either at the individual or at the dyadic level, probably because lactation, more than other social features, influenced the sow response. Moreover, it must be considered that the sows were closely related to each other—siblings or half-siblings—and this may have influenced the levels of affiliation, with individual frequencies of affiliation being quite similar across sows. Moreover, aggression can be linked to testosterone, which also in females (and not just in males) can antagonise the effect of pro-maternal hormones and increase aggressive behaviour [113,114,115]. These aspects are in line with our results showing higher levels of response from sows that exhibited lower frequencies of aggressive behaviour.

We expected that the highest frequencies of behavioural responses by sows would be associated with more intense and longer-lasting piglet isolation calls. Contrary to this assumption, our results show that proactive response levels are higher when isolation calls are characterised by lower intensity (lowest maximum dB), whereas there are no differences in the levels of reactive responses, and the duration of piglet isolation calls did not have a significant effect (Figure 7, assumption 4 from Table 3 not confirmed). In this regard, this aspect of piglets’ isolation calls seems to play a key role in eliciting the approach behaviour of sows towards infants in distress. This would seem to indicate that there may be characteristics of the isolation call that allow a piglet in distress to effectively communicate its state of need and attract the sow’s attention.

However, with respect to the response of sows, our results may offer interesting insights. Mothers may respond to isolation calls by regulating their own behaviour, and response behaviours are conserved among mammals, including humans. In order to explain this result, it is important to take into account that—from the point of view of social cognition—proactive responses may involve an additional cognitive step in that an uninvolved third party must first detect and process subject’s distress (caused by another subject or an external event) and then approach the distressed individual. Therefore, elements of complex regulation mechanisms may be present [116], as the uninvolved party takes the initiative and actively approaches the source of stress in order to possibly modify its own experience (intrinsic regulation) and/or the contacted party’s experience (extrinsic regulation).

Finally, during vocal production, emotions can influence the physiological structures underlying sound production and thus change the structure of the sound itself, including the duration of the vocalisation, amplitude, fundamental frequency, and energy distribution [14]. Therefore, other features of isolation calls could also have an effect on the response behaviour of sows, and it would be interesting to investigate this in future studies.

A possible limitation of this study may be related to the small sample size (if we consider the absolute number of individuals) and to differences in the age of lactating and non-lactating sows, so we encourage future studies to expand and/or replicate this investigation to possibly make inferences at the population or species level. On the other hand, this investigation was carried out under extensive, natural farming conditions, and in terms of study design, we managed to have a similar number of lactating and non-lactating (sexually mature) females exposed to the same environmental conditions in the same season (hence, the same climate), with the piglets all falling within the pre-weaning period. The perception and recognition of a state of anxiety in another individual could be further key factors, and we cannot exclude that the behavioural response observed by the sow might be the result of emotional contagion. From an adaptive point of view, all these behavioural aspects could contribute to increasing the survival probability of the offspring.

## 5. Conclusions

Hence, our study can add to our knowledge of how sows ecologically respond to distressed piglets. In particular, our results show increased levels of anxiety behaviour in sows, accompanied by increased responses to distressed piglets. Indeed, the higher levels of response in lactating females compared to non-mothers may be explained by the influence that maternal hormones could have on the behaviour of lactating sows by increasing their care activities and recovering/vigilance and by stimulating mother-offspring attachment [107,108,110,111,112]. In conclusion, the results of this study highlight the possible adaptive value of sows’ ability to recognise manifestations of others’ anxiety and consequently react appropriately.

## Figures and Tables

**Table 1:** List and characteristics of sows.

Sow	Sow Category	Age (Months)	Date of Delivery	Litter (Number of Piglets)	Trials as Mother	Trials as Non-Mother
3PB	Lactating female	18	2 January 2022	6	12	25
AAB	Lactating female	12	23 December 2021	3	12	25
AAG	Lactating female	12	15 January 2022	6	12	25
AAR	Lactating female	12	22 December 2021	8	12	25
SB	Lactating female	18	1 January 2022	8	12	25
SR *	Lactating female	18	15 January 2022	0	0	37
QQB	Non-lactating female	7	NA	NA	NA	37
QQR	Non-lactating female	7	NA	NA	NA	37
XXB	Non-lactating female	7	NA	NA	NA	37
XXR	Non-lactating female	7	NA	NA	NA	37

* SR lost her litter on the first day after birth but kept lactating piglets of other sows. NA: not applicable.

**Table 2:** Aggressive, affiliative, and anxiety-related behaviours of domestic pigs (*Sus scrofa*) considered in this study [29,50].

Category	Behavioural Pattern	Description
Anxiety-related behaviours	Body scratching/rubbing	A pig uses its legs or a substrate to rub parts of its body.
	Vacuum-chewing	A pig chews with an empty mouth.
	Head/Body shaking	A pig vigorously shakes its head and/or body.
	Yawning	A pig performs deep, long inhalation with an open mouth.
Aggressive behaviours	Aggressive lifting	A pig attempts to displace a fellow by lifting or levering it with its snout or head.
	Aggressive biting	A pig opens its mouth and closes its teeth tight on a fellow’s small piece of flesh, including its tail.
	Aggressive mounting	A pig forces a fellow to move away by rising upon its rear.
	Aggressive kicking	A pig projects of one or both hind limbs towards a fellow, striking it.
	Aggressive pushing	A pig presses its head, neck, shoulder, or body against a fellow, thus causing its movement.
	Aggressive chasing	A pig pursues a fellow, which flees.
	Aggressive head-knocking	A pig lurches or jerks its head, hitting a fellow.
	Fighting	Two pigs mutually push one another in a head-to-head orientation. The pattern can involve body-to-body rotation and/or aggressive mounting, lifting, biting, attempt biting, kicking, chasing, pushing, head knocking, and high-pitched vocalisation, with no interruption lasting more than 10 s.
Affiliative behaviours	Rest in contact	Two pigs sit or lay in contact with one another.
	Social touching	A pig touches a fellow with a paw or other body parts, except the nose/head.
	Nose–nose contact	A pig touches with its nose the nose of a fellow.
	Nose–body contact	A pig touches/pushes with its nose a body part of a fellow (excluding the nose).
	Head-over	A pig puts its head above the back of a fellow; rest in contact or body contact can then occur.

**Table 3:** The association between each GLMM analysis and specific assumptions under the general hypothesis that sows may respond to piglets’ isolation calls via arousal increase, with their response possibly varying depending on individual, perceptual, and social factors.

Sow Sample	Assumptions within the General Hypothesis	Analysis
All sows	1. We expected that isolation calls of piglets would cause: (1a) increased levels of anxiety-related behaviours in sows; (1b) increased frequency of behavioural responses; (1c) increased frequencies of responses in mothers of the piglet that emitted the isolation calls compared to non-mothers; (1d) a higher frequency of responses by sows showing lower levels of aggression.	GLMM_1_: Individual factors possibly affecting the occurrence of sow response to piglet vocalisation (N_cases_ = 370). Target variable: occurrence of the response (presence/absence) of the sow to the piglet’s isolation call. Fixed factors: (i) sow category (non-lactating female, lactating non-mother, lactating mother); (ii) sow aggressive level (non-top/top quartile). Further control (fixed) factors: (i) piglet age (days); (ii) piglet sex (male/female); (iii) distance between the vocalising piglet and sow (metres); (iv) isolation call duration category (non-top/top quartiles); (v) max decibel intensity of isolation call (non-top/top quartile); (vi) sow affiliation level (non-top/top quartile); (vii) number of trials for each sow (numeric). Random factors: sow and piglet identity.
Responding sows	2. We expected that more intense and longer-lasting piglet isolation calls might elicit a higher frequency of behavioural responses from the sows.	GLMM_2_: Perceptive factors possibly influencing the type of response (N_response_cases_ = 58). Target variable: type of the sow response to the piglet’s isolation call (proactive/reactive). Fixed factors: (i) isolation duration category (non-top/top quartile); and (ii) max decibel intensity of vocalisation (non-top/top quartile). Random factors: sow and piglet identity.
Non-mother sows	3. We expected that the sows would respond more to vocalisations emitted by piglets that were not their offspring when they had higher affiliation levels—and lower aggressive levels—with the mother of the vocalising piglet.	GLMM_3_: Possible influence of the relationship between the sow and the mother of the vocalising piglet on the response probability. (N_non_mother_cases_ = 320). Target variable: occurrence of the sow response (presence/absence). Fixed factors: (i) aggressive levels of the dyad non-mother and mother of the vocalising piglet (non-top/top quartile); (ii) affiliation levels of the same dyad (non-top/top quartile). Random factors: sow and piglet identity.
Lactating females	4. We expected to find higher levels of response in sows with higher lactation rates and duration.	GLMM_4_: Possible effect of lactation variables on the sow response (N_lactating_female_cases_ = 222). Target variable: occurrence of sow response (presence/absence). Fixed factors: lactation duration (non-top/top quartile); lactation frequency (non-top/top quartile). Random factors: sow and piglet identity.

**Table 4:** GLMMs results: influence of individual factors (GLMM_1_), relationship with the mother of the vocalising piglet (GLMM_3_), and lactation variables (GLMM_4_); influence of perceptual factors on the type of response (GLMM_2_).

Predictors	Estimates	SEM	CI_95_	Effect Size	χ2	*p*
GLMM_1_	n_cases_ = 370; full vs. null model: χ^2^ = 31.226; df = 10; *p* < 0.001					
Intercept	−2.961	0.946	−3.73, −1.56	^a^	^a^	^a^
Sow category (lactating mother) ^b^	1.912	0.630	0.68, 3.15	1.91	3.037	**0.002**
Sow category (non-mother) ^b^	2.859	0.743	1.40, 4.31	2.86	3.849	**<0.001**
Distance (from 1 to 15 m)	−0.077	0.047	−0.72, 0.07	−0.33	−1.631	0.103
Scream duration (long duration) ^b^	0.120	0.422	−0.30, 0.41	0.05	0.284	0.776
Piglets sex (female) ^b^	−0.645	0.379	−1.39, 0.10	−0.64	−1.703	0.089
Piglet age	0.020	0.024	−0.25, 0.62	0.19	0.844	0.399
Isolation call maximum intensity (high intensity) ^b^	−0.700	0.453	−1.59, 0.19	−0.70	−1.545	0.122
Sow aggression frequency (high levels) ^b^	−0.886	0.433	−1.73, −0.04	−0.89	−2.045	**0.041**
Sow affiliation frequency (high levels) ^b^	0.537	0.724	−0.88, 1.96	0.54	0.742	0.458
Sow trial number	0.003	0.019	−0.35, 0.42	0.03	0.174	0.862
GLMM_2_	n_cases_ = 58; full vs. null model: χ^2^ = 6.390; df = 2; *p* = 0.041					
Intercept	1.533	0.432	0.69, 2.38	^a^	^a^	^a^
Scream duration (long duration) ^b^	−0.233	0.695	−1.60, 1.13	−0.23	−0.335	0.738
Isolation call maximum intensity (high intensity) ^b^	−1.590	0.652	−2.87, −0.31	−1.59	−2.439	**0.015**
GLMM_3_	n_cases_ = 320; full vs. null model: χ^2^ = 1.323; df = 2; *p* = 0.516					
GLMM_4_	n_cases_ = 222; full vs. null model: χ^2^ = 0.325; df = 2; *p* = 0.850					

Note: Random factor: Piglet identity and Sow identity (GLMM_1_, GLMM_2_, GLMM_3_, GLMM_4_). Bold values indicate *p* < 0.05. Abbreviations: CI, confidence interval; SEM, standard error. ^a^ Not shown as not having a meaningful interpretation. ^b^ These predictors were dummy-coded, with the reference category as follows: Sow category: “lactating non-mother”; Piglet sex: “male”; Scream duration: “short duration”; Isolation call maximum intensity: “low intensity”; Sow aggression frequency: “low levels”; Sow affiliation frequency: “low levels”.

## Data Availability

The data that supports the findings of this study are available in the Supplementary Materials of this article.

## Supplementary Materials

The following supporting information can be downloaded at: https://www.mdpi.com/article/10.3390/ani13142261/s1, Data S1: data sheets; Video S1: proactive response; Figure S1: GLMM_s_ residuals plots.
