# Supplementary figures and images for: Sows’ Responses to Piglets in Distress: An Experimental Investigation in a Natural Setting
Source: Animals (Basel). 2023 Jul 10;13(14):2261. doi: 10.3390/ani13142261 (PMC10376744; doi:10.3390/ani13142261)

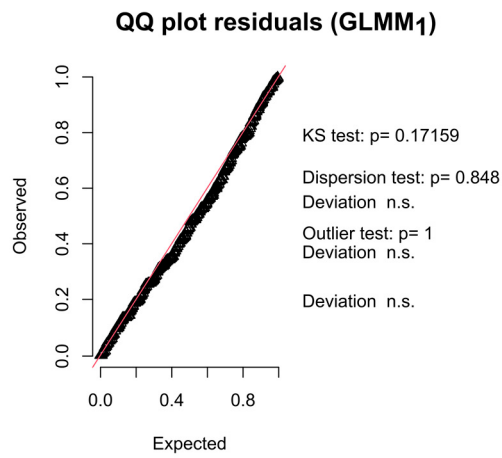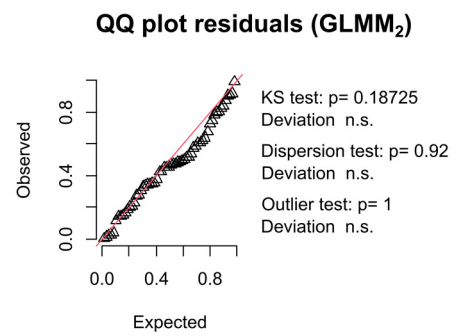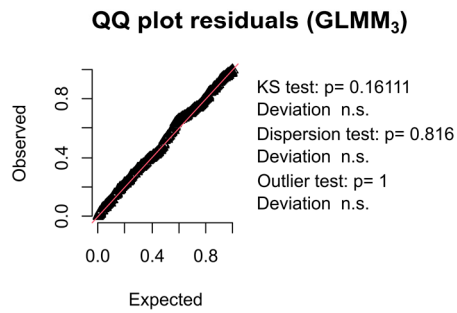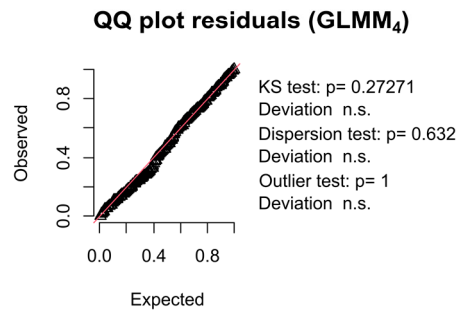

**Figure S1.** GLMMs residuals plots.

Supplement: Supplementary file 1 [file animals-13-02261-s001.zip › Figure S1.pdf]
